# Pulmonary injury associated with spray of a water-based nano-sized waterproofing product: a case study

**DOI:** 10.1186/s12995-017-0180-7

**Published:** 2017-12-08

**Authors:** Paul T. J. Scheepers, Lucie Masen-Poos, Frits G. B. G. J. van Rooy, Arné Oerlemans, Eline van Daalen, Robbert Cremers, Hera Lichtenbeld, Bonne Biesma, Jorid B. Sørli, Ismo K. Koponen, Søren Thor Larsen, Peder Wolkoff, Asger W. Nørgaard

**Affiliations:** 1 Research Lab Molecular Epidemiology, Radboud Institute for Health Sciences, Radboudumc, PO Box 9101, 6500 HB Nijmegen, The Netherlands; 2 Department of Lung Diseases, Jeroen Bosch Hospital, ’s-Hertogenbosch, The Netherlands; 3 Arbo Unie Expert Centre for Chemical Risk Management, Utrecht, The Netherlands; 4 Oxility BV, Best, The Netherlands; 5 The National Research Centre for the Working Environment, Copenhagen, Denmark; 6 Present address: Ministry of Social Affairs and Employment, The Hague, The Netherlands; 7 Present address: Witteveen+Bos Consulting, The Hague, The Netherlands

**Keywords:** Chemical pneumonitis, Exposure reconstruction, Inhalation injury, Occupational accident, Waterproofing

## Abstract

**Background:**

In most reported cases of lung trauma with water proofing products, volatile organic compounds (VOC) have a prominent role. Here we report on a case involving ten workers exposed to a sprayed product containing nanoparticles in a water solution with only a few percent VOC.

**Case presentation:**

Ten workers suffered from respiratory symptoms following spray impregnation of hardwood furniture using a waterproofing product that contained positively charged fluorinated acrylate copolymer solid cores with a median diameter of 70 nm (1.3 w%) in aqueous suspension with 3.3 w% VOC and 0.3 w% quaternary ammonium. The worker who applied one liter of the product in a wood workshop, using an air mix spray gun, did not report any health complaints. Another worker, who entered the workshop 3 h later and had rolled and smoked two cigarettes, was hospitalized with severe chemical pneumonitis. A chest X-ray (CXR) showed bilateral infiltrative impairment in the lower lobe regions. On the next day a second CXR showed increased patchiness marking in all fields. A high-resolution Computer Tomography (CT)-scan demonstrated extensive bilateral areas of ground-glass opacities predominantly in the lower regions of the upper lobes, the right middle lobe and the apical regions of the lower lobes, compatible with severe chemical pneumonitis. On the following morning, nine workers in an adjacent workplace in the same building, experienced dry cough, chest tightness and substernal pain upon physical exercise. Reconstruction of the spray application in a climate chamber confirmed trimethyl silanol, glycol ethers and fluoroalkenes in the gas phase. Immediately after the spray application, aerosols were observed at a maximum concentration of 6.3 × 10^4^ cm^−3^. Mass concentrations were 0.095 and 10 mg/m^3^ in the size ranges 5.6-560 nm and 0.22-30 μm, respectively, decreasing to less than 10 μg/m^3^ in both size ranges after 15 h.

**Conclusion:**

The hospitalized worker had smoked cigarettes contaminated with fluoropolymers which is a plausible explanation for the lung trauma. Respiratory symptoms in the nine workers may be caused by inhalation of particles that became airborne by resuspension from surfaces when workers entered the adjacent workplace the next day. A contribution from VOC appears less likely because measurements and modelling showed that concentrations in the mg/m^3^ range could have occurred only if the building was assumed to be completely airtight.

**Electronic supplementary material:**

The online version of this article (10.1186/s12995-017-0180-7) contains supplementary material, which is available to authorized users.

## Background

Waterproofing products are used to coat textile fabric, leather or solid surfaces to ensure water and dirt resistance. These products usually contain three key components: a water repellent (active compound), a solvent and a propellant (if the product is canned). The water repellent is generally a mixture of siloxanes or acrylate polymers – both with fluorocarbon or hydrofluorcarbon chains. In products marketed before 2000, the solvent used to be an aliphatic hydrocarbon and sometimes chlorinated or cyclic hydrocarbons were used [1–3]. Nowadays, aqueous mixtures of glycols and glycolethers are often used as solvents [4, 5]. Propellants are usually C_3_-C_4_-alkanes or CO_2_ (Additional file 1: Figure S2).

Over the past 20 years, different health effects from the use of waterproofing agents have been described in approximately 20 reports involving exposure of over 200 individuals. The first report is from 1983 and described a case in the US of a consumer who used a 1,1,1-trichloroethane-based product [1]. Other reports describe cases with a variety of products, containing trimethylpentane solvent and paraffins [6], ethyl acetate and n-heptane [3] and petroleum hydrocarbons [7]. Most case-reports did not describe the product composition [2, 8–12]. Overall, these cases report mostly local effects on the airways and dyspnea with dry cough as the common denominator. However, tachypnea, tachycardia and mild cyanosis was also reported [6, 7, 10, 13, 14]. Only a few cases reported a slight increase of body temperature and only one study reported neurotoxic signs [5]. There is wide consensus that the use of propellants such as propane and butane isomers does not represent a hazard [2, 15]. In most case reports, the active fluorinated compounds were implicated as the agents responsible for the acute pulmonary response [2, 3, 7, 14, 16]. Only the first reported case of an intoxication [1] suggested the organic solvent 1,1,1-trichloroethane as the primary cause of the pulmonary complaints. More recent reports suggest involvement of an organic solvent to explain some of the specific local and systemic effects [5]. A change of isopropanol to heptane as a solvent was implicated as a probable cause of acute respiratory symptoms in a Swiss incident, but in this case the type of fluorocarbon resin used was also changed, along with the solvent [4]. In an incident in a supermarket alkylsiloxanes and C_9_-C_13_ alkanes were involved [17]. Recently, two studies associated the airway toxicity of a perfluorinated silane with the number of hydroxyl groups in addition to the properties of the used solvent [18, 19].

Most of the aforementioned reports suggest that health complaints arise from inhalation of spray aerosols. Some studies suggest that the particle size and number characteristics of inhaled sprays could play a role in the acute pulmonary toxicity [2, 5, 20, 21]. Waterproofing agents that interact with the thin film of liquid covering the alveoli implicate lung surfactant deterioration as the cause of the respiratory problems [6, 17, 19–28]. Some authors have also made reference to “polymer fume fever”, that has been observed after inhalation of pyrolysis products of e.g., polytetrafluoroethylene (Teflon) [2, 6, 29]. Two case reports described the use of waterproofing product in conjunction with smoking [11, 12].

In this study we describe the results from two reconstructions of the spraying process of wood coating with the purpose of identifying the potential causative agents.

## Case presentation

### Product application

The incident occurred in an occupational setting and relates to a self-employed carpenter (person A). Co-exposures involved his wife (person B) and a hired mechanic (person C). At 17.00 h on the day of the incident, a carpenter (person A, a non-smoking male of 47 y) treated two tabletops (approximate surface area of 2 m^2^) with a waterproofing product (cf Table 1 for information on the composition). The spray application was performed in a wood workshop for the production of hardwood furniture with a volume of ca. 2.600 m^3^ (h x l x w = 4 × 32.5 × 20 m) without mechanical ventilation (Additional file 1: Figure S1). One liter of the product was sprayed undiluted at a distance of approximately 0.5 m from the surface over a period of 4 min using an air mix spray gun (Eminex type E31 EHT M 01 with a 1.80 mm nozzle diameter, Eminent, Oss, NL) at a pressure of 2 bar. No gloves or respiratory protection was used. No other chemicals than the waterproofing product were used. During spraying, person A did not report any smell. Around 18:15 person B, the wife of the carpenter (non-smoker, 43 y) arrived and spent approximately 15 min in the workshop (see Additional file 1: Table S1 for a complete timeline). Both person A and person B reported not to have seen any mist or noticed any unusual smell nor did they experience any health problems. At 19:45, person C, a mechanic (male, smoker of 5-20 cigarettes/day, 40 years of age) arrived to do some repair work. He spent about 10 min in the room where the tables had been sprayed. He also did not notice any spray mist or smell and was not wearing any skin or respiratory protection. Around 20:30 he spent another 15 min in the workshop repairing a milling machine. He did not have direct contact with the treated table tops. He picked up and used a vernier caliper that was lying around (at least) 10 m from the treated tabletops. During his work he did not wear gloves. He did not remember if his hands were somehow contaminated when returning to the office. Around 22:00 he rolled two cigarettes. He did not notice any strange smell or taste when smoking these cigarettes outside. At 23:15 he experienced serious dyspnea and asphyxiation (perceived as if ‘drowning’) and reported to the emergency room of the local hospital.

**Table 1:** Formulation of the waterproofing product as reported by the producer

Component	CAS^a^	Fraction (w%)
Water	7732-18-5	95.3
Fluoroalkyl ethyl acrylate co-polymer	–	1.3
Propylene glycol	504-63-2	1.3
Alcohols, C12-C14-secondary, ethoxylated	84133-50-6	1.3
Dipropylene glycol monomethyl ether	34590-94-8	0.3
Polyethylene glycol trimethylnonyl ether	84133-50-6	0.3
Quaternary ammonium compounds, bis(hydrogenated tallow alkyl)dimethyl chlorides	61789-80-8	0.3
Polyether (not specified)	–	0.1

^a^Chemical identification corresponding to CAS number reported in ChemIndex list of synonyms http://ccinfoweb.ccohs.ca/chemindex/search.html)

### Secondary exposure

In the same building, next to the wood workshop, a mail sorting and distribution centre was located. This workshop (h x l x w = 4 × 30 × 20 m) had also no forced ventilation. There was an open air (space) connection near the roof of 0.1 m over a distance of about 20 m in a brick wall between the wood workshop and the mail sorting and distribution centre (see Additional file 1: Figure S1). The first worker who entered the building at 7:45 on the day after the application of the waterproofing spray, did not observe any mist or unusual smell. But around 8:30 he started to cough and by 10:00 another eight workers reported a painful and persistent dry cough. Some of them also reported chest tightness and sub-sternal chest pain upon physical exercise. Neither the employer nor the workers had been informed about the incident that had occurred in the wood workshop on the day before. On the morning of the second day after the incident the workers still suffered from cough; however, anamnesis and physical examination of four workers by an occupational physician did not reveal any abnormalities of respiratory function. During the following days the workers did not feel well, reporting fatigue and weakness. After a week all workers had fully recovered and had returned to their work. The outdoor weather conditions on the day of the incident and the day of the second exposure are presented in Additional file 1: Table S2.

### Physical examination

**Fig. 1:**
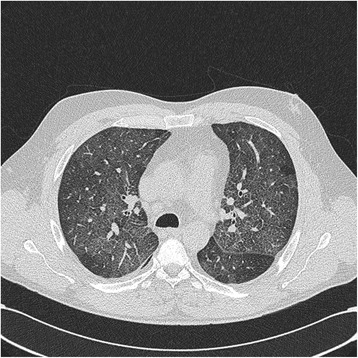
HRCT of thorax on the second day of hospitalization showed extensive bilateral areas of ground-glass opacities predominantly in the lower regions of the upper lobes, the right middle lobe and the apical regions of the lower lobes, compatible with severe chemical pneumonitis

Person C had no history of respiratory disease or pre-existing sensitivity, asthma or allergy. Upon arrival in the emergency room at 23:30 he suffered from dyspnea, cough and produced clear sputum. He felt cold, reported impaired vision, his hands were shaking and he also reported a tingling sensation in his extremities. Blood pressure was 125/61 mmHg and his pulse rate was 105/min. His body temperature was 39.0 °C that normalized within 6 h. Blood saturation was 93% on a non-rebreathing oxygen mask (15 L/min) and decreased to 60% rapidly without respiratory support. Heart sounds were normal and airway examination by stethoscope indicated vesicular respiratory sound on both sides. Analysis of a venous blood sample showed a leukocytosis (19.1·109/L) and an elevated C-reactive protein content (CRP, 57 mg/L), rising to a maximum of 112 mg/L on the third day of hospital admission. A chest X-ray (CXR) was taken at 2:00 in the night of admission (not presented). A high resolution computed tomography (HRCT) thorax (Fig. 1) indicated extensive bilateral areas of ground-glass opacities and predominantly in the lower regions of the upper lobes, the right middle lobe and the apical regions of the lower lobes. There were no signs of honeycombing. An aspiration pneumonitis as most probable course was suggested by the radiologist.

No antibiotics were given as, from the start, a pneumonitis due to chemical exposure was found to be the most likely diagnosis. During his stay at the emergency unit and afterwards at the pulmonology ward the Netherlands poison centre (NVIC) was contacted several times. Treatment was started and prednisolone was tapered after consultation of the poison centre. Person C was treated with oxygen, Salbutamol (2.5 mg) and Atrovent (500 μg) by inhalation and received 50 mg of prednisone i.v. four times a day. The patient remained hypoxic with desaturation upon physical exercise, for a week. Recovery was slow, but oxygen supply could gradually be reduced. The patient was dismissed from the hospital five days after the incident and then followed-up in the outpatient clinic for another three weeks. Lung function showed a minimal obstruction: FVC 3.99 L (91% predicted), FEV1 2.89 L (80% predicted). FEV1/FVC 73.3 (92% predicted), and TLCO 7.56 mmol/min/kPa (74% predicted). Treatment with prednisone was gradually reduced and terminated within two weeks at the outpatient clinic. Two weeks after admission the patient did not report any airway complaints. The blood oxygen saturation was 96% without oxygen supplement and the CXR appeared normalized (not presented).

## Methods

### Chemicals

2-(2-Butoxyethoxy) ethyl acetate, decamethyl tetrasiloxane, di(propylene glycol) methyl ether (mixture of isomers), 2-ethylhexyl acrylate, hexamethyl disiloxane, methanol, 2-(2-methoxypropoxy)-1-propanol, 1H,1H,2H-perfluoro-1-decene, 1H,1H,2H,2H-perfluorodecyl acrylate, 1H,1H,2H,2H-perfluoro-1-octanol, toluene, trimethyl silanol were obtained from Sigma Aldrich (Brøndby, Denmark). 1H,1H,2H-perfluoro-1-tetradecene was obtained from Synquest Laboratories, Alachua, FL. All chemicals were of the highest available purity (98-99.9%).

### Waterproofing product

The label of the product indicated it was water-based. According to the user’s instructions it would make wooden surfaces water and dirt resistant and explain how it should be applied. The product was advertised for surface treatment of hardwood furniture (for both indoor and outdoor use). The label did not contain symbols referring to any health or environmental hazards, but the product contained the following phrases (translated from Dutch/French): ‘Keep out of range of children; prevent direct contact with skin and eyes; use only in a well-ventilated area; store in closed bottle; do not induce vomiting if swallowed and consult a physician and show the package and label; we do not accept liability in the case of wrong use; attention: store frost-free’. The label did not provide further information about the composition, e.g. fluorinated compounds or nanoparticles. The manufacturer provided information about the formulation of the product to the authors (Table 1). On the label, instructions were given to rub the product on the surface using a cloth. However, in this case the product was sprayed by use of a spray gun.

Analysis of the product showed presence of nano-sized spheres with a median diameter of ca. 72 nm, with strong hydrophobic properties and with a slight positive charge (zeta potential of +31.3 mV, see Additional file 1: Figure S2-S5). These particles formed a stable suspension in water with a negligible tendency to form clusters. The particles were chemically characterized as water-free solid organic silica cores, with a soft shell consisting of tri-block-copolymers containing perfluoroalkyl acrylate, in addition to polyethylene oxide and polypropylene oxide, and presumably end-capped with ethylene oxide.

### Reconstruction of spray incident

In order to obtain information about the emission of volatile organic compounds (VOC) and aerosols during and after application of the product, spray tests were carried out under different test conditions in two different test chambers (Experiments 1 and 2). Experiment 1 was carried out at 6-fold the concentration of the incident; Experiment 2 at 46-fold higher concentration for the evaluation of larger particles and collection of particle samples for further analysis (not presented here). In both experiments, the spray gun (Eminex type E31 EHT M 01, Eminent, Oss, NL) was equipped with similar nozzles and operated at the same spray pressure (2.0 Bar) as the one originally used in the wood workshop on the day of the incident.

### Experiment 1

In this experiment, 47 g of product was sprayed in a uniform layer on a 0.6 m^2^ untreated plywood surface placed inside a climate steel chamber (h x l x w = 2.29 × 3.46 × 2.56 m) with an ante-chamber (2.72 m^3^) as inner entrance. The experiment was carried at a ventilation, temperature and relative humidity of 0.08 ± 0.03 h^−1^, 22 ± 2 °C and 45 ± 5%, respectively. Immediately after the start of the experiment, three mixing fans were turned on for 60 s in order to ensure a homogeneous distribution of aerosols and VOC. The fans were placed in three corners on the floor and at 5 cm from the chamber wall. VOC were sampled through a 10 mm stainless steel sampling manifold placed at a height of 1.0 m from the floor and ca. 1.2 m from the spray position; air was sampled at 5 cm from the inner chamber wall. During the first 5 h, samples were taken in duplicate at 10-30 min intervals, starting the first sampling event 1 min after the spray application was initiated. Additional samples were taken after 23 and 25.5 h, following the start of the spray application, respectively. The time of sampling is given as the midpoint between start and end of each sampling period. VOC data are reported as mean of duplicates, corrected for chamber background air and rounded to the nearest integer. VOC were sampled on clean Tenax TA (60-80 mesh) adsorbent tubes (200 mg) with a sampling time of 10 min at 100 mL/min, using calibrated pumps (Gillian Gilair 5, Sensidyne, US). The Tenax TA tubes were analyzed on a Perkin Elmer Turbo Matrix 350 thermal desorber (TD) coupled to a Bruker SCION TQ GC-MS system (Bruker Daltonics, Bremen, DE). Tube desorption was carried out at 275 °C for 20 min and the low and high temperatures of the cryo trap were −20 °C and 280 °C, respectively. Separation was performed on a 30 m GC column with 0.25 mm internal diameter and 0.25 μm film thickness (type VF-5MS, Agilent Technologies, Santa Clara, US). The oven program was as follows: 50 °C for 4 min, ramp 1: 4 °C/min to 120 °C, ramp 2: 50 °C/min to 250 °C, hold for 2 min. Helium was used as carrier gas at an inlet pressure of 0.97 bar (1.5 mL/min). The mass spectrometer was operated in SIM/scan mode using either electron ionization or chemical ionization with methane (5.0) as ionization gas. Argon of ultrahigh purity (99.999%) was used for collision induced dissociation (CID) experiments. Valves, transfer lines and ion source were kept at 270 °C. Six-point calibration was applied (r^2^ > 0.99) using authentic standards in methanol. Identification of observed VOC was based on retention time and mass spectra of authentic standards, when applicable, in addition to library search [29], chemical ionization and CID.

Number size distribution measurements were conducted using a TSI Model 3091 Fast Mobility Particle Sizer (TSI, Shoreview, NM). The instrument was operated at 1-s time resolution in a measurement range of 5.6-560 nm. An optical particle spectrometer Grimm 1.109 (Grimm Aerosoltechnik, Ainring, Germany) was used to measure the number size distribution from 0.25 to 32 μm at 6 s time resolution (count distribution mode). Total number concentration was integrated from the number size distribution. Spherical SOA particles with a density of 1.0 g/ml was assumed for mass calculations. In addition, a density of 2.6 g/ml was used for comparison of mass results with experiment 2 measurements where an environmental mode was used (in the environmental mode a default density of 2.6 g/ml was also used to calculate mass concentration).

### Experiment 2

In this experiment, ca. 0.7 l of the product was sprayed ‘mid air’ inside a 40 m^3^ paint booth (h x l x w = 4.0 × 4.0 × 2.5 m) during a period of 10 min. During the last 2 min of spraying, the spray booth was turned off to allow the particles to remain in the booth and to describe the time-resolved post-spraying changes in particle size. Aerosol mass size distribution spectra in the size range from 0.25 to 32 μm were measured using a Grimm 1.109 operating at a time resolution of 6 min. The instrument was set to occupational mode (PM-10, PM-2.5, PM-1.0 in μg/m^3^). A nephelometer (type IV Hazdust, Environmental Devices, Plaistow, NH, USA), equipped with a cyclone pre-seperator, was used for collection of the thoracic fraction (equivalent to PM-10) at a 10-s time resolution.

Filter samples were collected twice; the first period of 0-50 min after the start of application and a second period of 1-16 h after application. Inhalable dust was collected using an IOM sampler and a calibrated personal air sampling pump (A.P. Buck, Orlando, Florida, USA) at 2.0 L/min. A similar set-up was used to collect respirable particles by a Cassella cyclone (Cassella Measurement, Bedford, UK) at 1.9 L/min. Thoracic dust and respirable dust fractions were collected using PM-10 and PM-2.5 Harvard impactors, respectively, at 10.0 L/min. The filter samples were collected 2.0 m from the spray application at height of 1.2 m from the floor. All filters used were Teflon membrane filters (Sartorius, Göttingen, Germany).

### Modelling

A two compartment model was constructed using Simulink/MatLab. This model was used to estimate the time pattern of VOC in the wood workshop and the mail sorting and distribution centre.

The air concentrations of VOC were modelled using the measurement data from experiment 1. The modelling was carried out by use of the data from total glycol ether which was by far the most abundant of measured VOCs. The initial concentration of the glycol ethers at the start of the spray application in the wood workshop was calculated to be 46 mg/m^3^, using a standard spray scenario of the exposure model ConsExpo 5.0 (www.rivm.nl/en/Topics/C/ConsExpo). The inter-compartment flow (Qi) in the open air connection with the dimensions of 20 × 0.1 m (2.0 m^2^) was estimated to be 7200 m^3^/h for an air speed of 1 m/s (1 m/s × 2.0 m^2^ = 2 m^3^/s = 7200 m^3^/h). We then estimated the concentration in the wood workshop over time by adopting an algorithm used by Vernez et al. [30]:

1 $$ {C}_1=\int \frac{1}{V_1}\ \left({Qi}^{\ast }{C}_2-{Qi}^{\ast }{C}_1-{Q}^{\ast }{C}_1\right) $$

where C_1_ is the concentration in the wood workshop (initial concentration 46 mg/m^3^),

C_2_ is the concentration in the mail sorting centre (initial concentration 0 mg/m^3^),

V_1_ is the volume of the wood workshop (2600 m^3^),

*Q* is the air exchange rate with outdoor air (0.08 h^−1^, 1.0 h^−1^ and 2.5 h^−1^),

*Q*_*i*_ is the inter-compartment flow (7200 m^3^/h).

A second equation was used to calculate the concentrations over time in the mail sorting centre:

2 $$ {C}_2=\int \frac{1}{V_2}\ \left({Qi}^{\ast }{C}_1-{Qi}^{\ast }{C}_2-{Q}^{\ast }{C}_2\right) $$

where C_1_ is the concentration in the wood workshop (initial concentration 46 mg/m^3^),

V_2_ is the volume of the mail sorting centre (2400 m^3^),

*C*_*2*_, *Q* and Qi are the same as in eq. (1).

The initial concentration in the mail sorting centre was assumed to be zero. As the complaints of the mail workers started at 8:00 am the next morning, we calculated the concentration at 15 h after the start of the spray event. We modelled three different air exchange rates. The first value of 0.08 h^−1^ is the air exchange rate of the measuring chamber and corresponds to a more or less airtight building. The other two values represent more realistic values to describe the type of naturally ventilated industrial building that is usually not very air tight, a low estimate of 1.0 h^−1^, corresponding to a low natural ventilation in a situation with low wind speed and closed doors and a high value of 2.5 h^−1^ to a moderately ventilated building with doors opened and at moderate wind velocity conditions. The real situation was probably in the range between these two conditions.

## Results

The concentration time-pattern following the spray application was followed in two separate experiments. Experiment 1 describes the results of a small-scale reconstruction in a measuring room, using only a small amount of product and focuses on submicron particles and VOC. Experiment 2 describes the use of a larger volume of the product in a spray booth. In this experiment the thoracic and respirable fractions were measured by optical instruments and filter sampling. The results of both experiments will be discussed below.

### Experiment 1

**Fig. 2:**
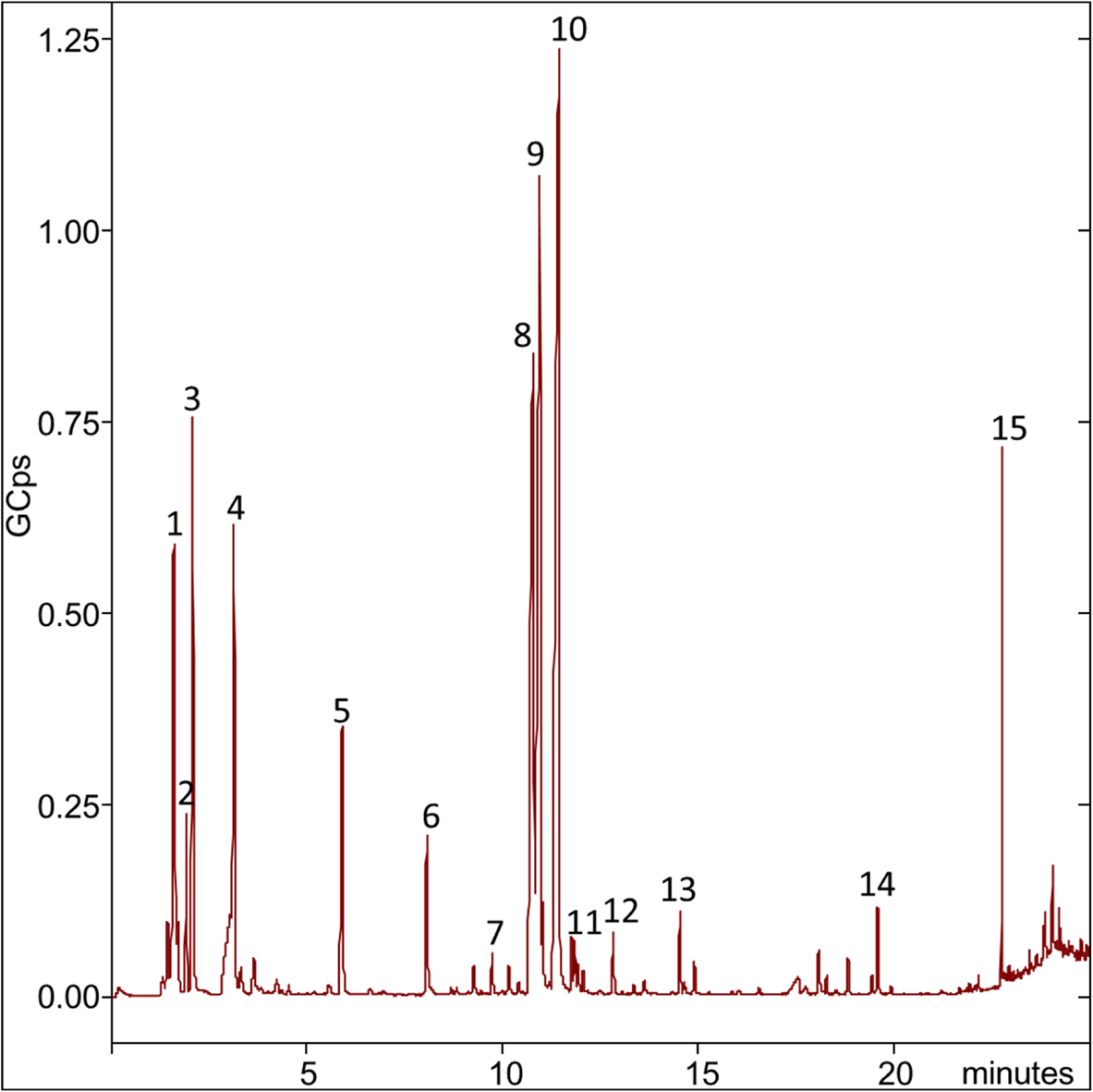
Total ion chromatogram (TIC) showing VOCs emitted to the gas phase 5 min after spraying 47 g of the product on a 0.6 m^2^ untreated wooden surface in Experiment 1 (see Table 2)

**Fig. 3:**
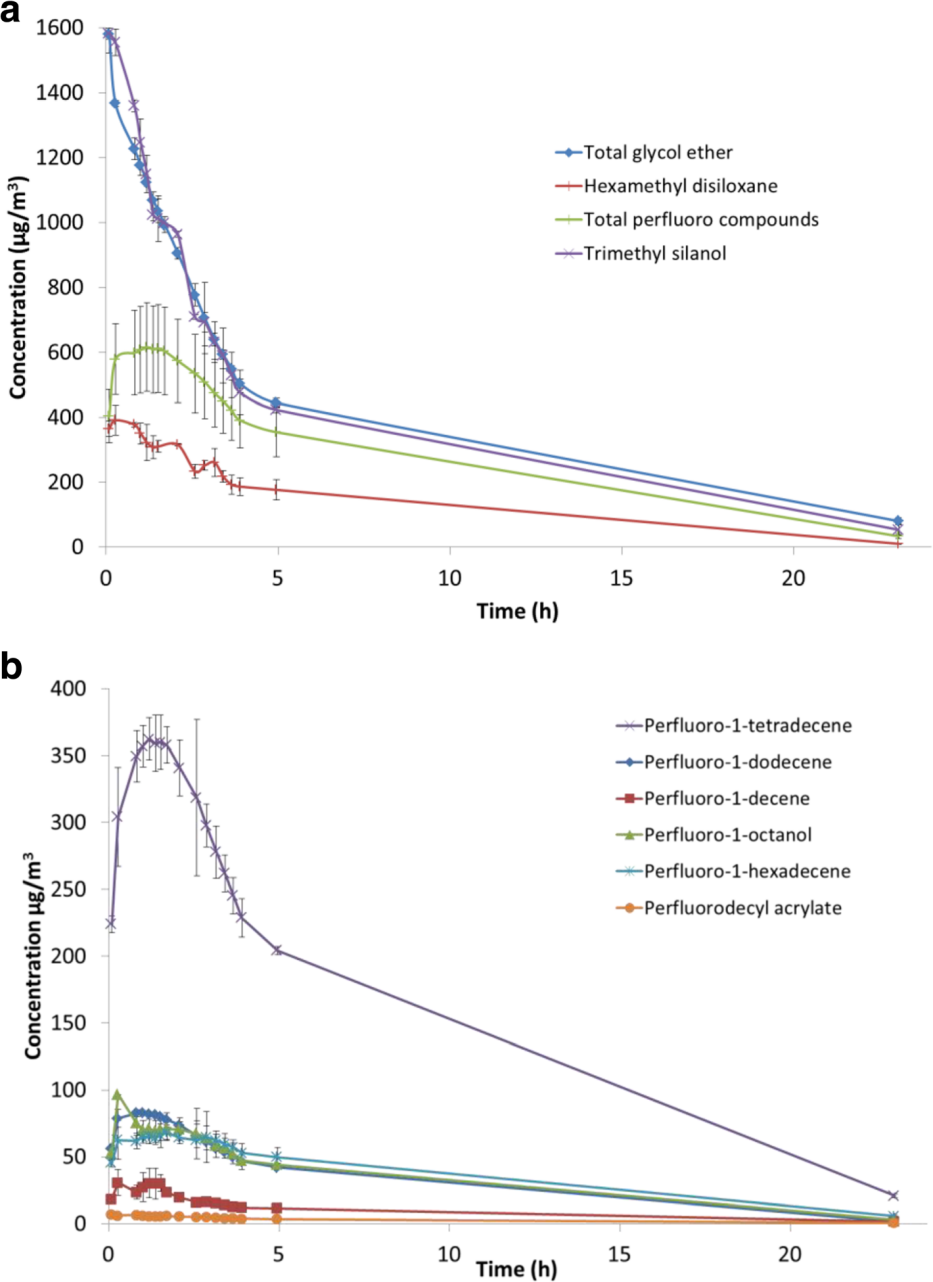
Time-concentration profiles of trimethyl silanol (peak 1) total glycol ether (peaks 8-10), total fluoro compounds (peaks 2, 4-7 and 13) and hexamethyl disiloxane (peak 3) (**a**); time-concentration profiles of fluoro compounds (**b**)

Use of the wood impregnation product resulted in an instant release of VOC, mainly trimethyl silanol, glycol ethers, fluoroalkyl compounds and a siloxane (see Table 2 and Figs 2 and 3). Trimethyl silanol, the glycol ethers and the siloxane reached maximum concentrations of 1.6 mg/m^3^, 1.6 mg/m^3^ and 0.4 mg/m^3^, respectively, within the first ca. 15 min after the spray event (Fig. 3a). Hereafter, the compounds decayed to concentrations of 9 μg/m^3^, 80 μg/m^3^ and 10 μg/m^3^, respectively, after 23 h. The concentration of the fluoroalkyl compounds (Fig. 3a and b) were dominated by 1H,1H,2H-perfluoro tetradecene and peaked between 1 and 1.7 h after the spray event. After a plateau, lasting about 1 h, all fluoroalkyl compounds decayed from a total maximum concentration of 0.6 to 0.03 mg/m^3^, after 23 h.

**Table 2:** Compounds identified in the gas phase after use of the wood impregnation product in Scenario 1. C_max_ and T_max_ are the maximum concentration and time of the maximum concentration, respectively

Peak	T_r_ (min)	Compound	CAS	Max conc. (μg/m^3^)	Max conc. (μg/m^3^ per g product)	T_max_ (min)	Boiling point (°C)	Vapor pressure (mbar)
1	1.7	Trimethyl silanol	1066-40-6	1582	33.7	5	99	21.0^c^
2	2.0	1H,1H,2H-perfluoro-1-decene	21652-58-4	31	0.7	16	146	8.5^d,e^
3	2.2	Hexamethyl disiloxane	107-46-0	392	8.3	16	100	56^d^
4	3.1	1H,1H,2H-perfluoro-1-dodecene^a^	30389-25-4	83^b^	1.8	60	202	1.3^d,e^
5	5.8	1H,1H,2H-perfluoro-1-tetradecene	67103-05-3	363	7.7	71	253	0.3^d,e^
6	8.0	1H,1H,2H,2H-perfluoro-1-octanol	647-42-7	97	2.1	16	90	0.5^d,e^
7	9.7	1H,1H,2H-perfluoro-1-hexadecene^a^	–	68^b^	1.4	102	–	–
8-10	10.6-11.3	Di(propylene glycol) methyl ether, mixture of isomers	34590-94-8	1581	33.7	5	203	0.13^d,e^
11	11.7	2-(2-methoxypropoxy)-1-propanol	13588-28-8	2	<0.1	5	190	0.67^d^
12	12.8	Decamethyltetrasiloxane	141-62-8	18	0.4	49	235^e^	0.13^d,e^
13	14.5	1H,1H,2H,2H-perfluorodecyl acrylate	27905-45-9	7	0.1	5	268	0.13^d,e^
14	19.5	2-ethylhexyl acrylate	103-11-7	8	0.2	16	233	0.13^d,e^
15	22	2-(2-butoxyethoxy)ethyl acetate	124-17-4	135	2.9	5	232^e^	0.13^d,e^

^a^) No standard; ^b^) 1H,1H,2H-Perfluoro-1-decene equivalents; ^c^) at 20 °C; ^d^) at 25 °C; ^e^) predicted values ACD/labs

**Fig. 4:**
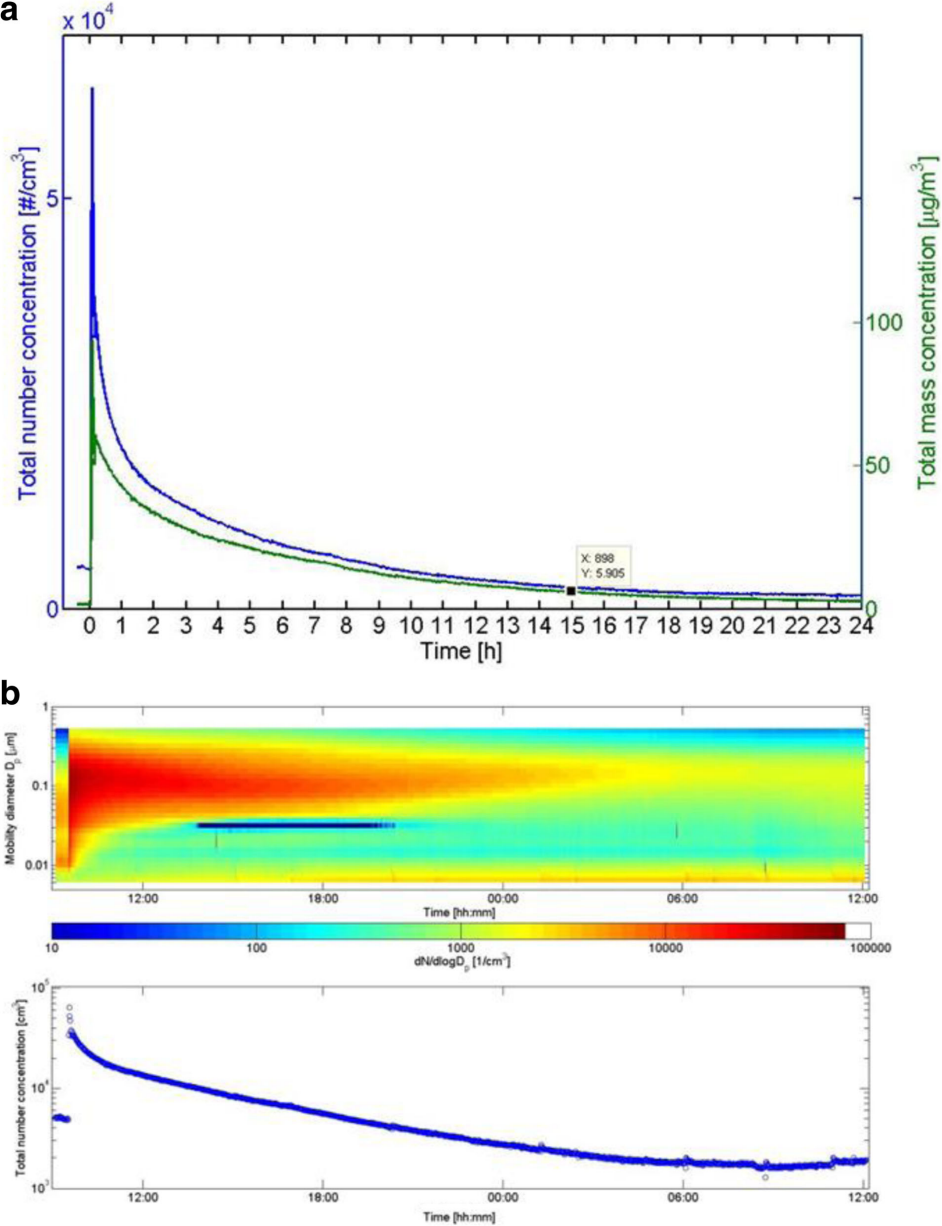
**a** Total number and mass concentration (density 1) calculated from the FMPS measurements in Scenario 1; *t* = 0 refers to the start of spraying. **b** Particle number size distribution and particle number concentration as the function of time

Figure 4a shows the number and mass concentration-time pattern for particles below 560 nm. An increase in the total number concentrations from 5.0 to 63 × 10^3^ cm^−3^ was observed immediately after the spray event. During the next 3 min, the particle number concentration about halved, followed by a slower decay, presumably due to wall losses and ventilation. The concentration returned to the initial background in 10 h after spraying.

The mass concentration increased from 5 to 95 μg/m^3^ during the first 2 min after the spray application, followed by a sharp decrease to 57 μg/m^3^ after 4 min. This was followed by a decay due to chamber ventilation, resulting in a concentration corresponding to the pre-experiment value of ca. 2 μg/m^3^, after ca. 24 h. The mass concentration did not decrease as fast as the number concentration during the first 1 h. Figure 4b presents the evolution of the number size distribution over 24 h with 1 min time resolution. The initial particle number size mode shows a peak around 100 nm that appears relatively stable during the entire 24 h measurement period.

### Experiment 2

**Fig. 5:**
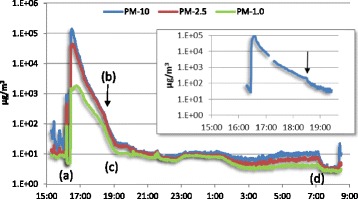
Concentration of three particle fractions in μg/m^3^ on a timescale representing local clock time during experiment 2. The inset shows the simultaneous registration of the concentration of the thoracic fraction (PM-10). (**a**) Paint booth ventilation turned on at 15:50 h; (**b**) increased ventilation due to a thunderstorm past over Nijmegen between 18:30 and 19:00 h causing a sudden decrease in dust concentration (see inset); (**c**) The ventilation system was shut off at 19:00. (**d**) The ventilation system is turned on at 07:00 h the next morning

The mass concentration profiles of aerosols are shown for PM-10, PM-2.5 and PM-1.0 in Fig. 5. When turning on the ventilation of the booth, the particle concentration dropped sharply and increased again when the spraying started. After shutting down the ventilation system at 16:25, the concentration of PM-10 peaked 16 min after spraying started at about 100 mg/m^3^ at 16:36; this was confirmed by the Hazdust measurement (inset in Fig. 5). The particle concentration gradually decreased due to the room ventilation of the building until this system was automatically shut off at 19:00 h. This time point matches the delay of ~2.5 h after the spray application when worker C was first exposed. At this time point the concentrations of PM-10, PM-2.5 and PM-1.0 were 32, 26 and 14 μg/m^3^, respectively. Over the period between 20:00 h and 1:00 h the three aforementioned PM fractions had very similar time-weighted average concentrations of 10, 9 and 8 μg/m^3^, respectively. The concentrations of PM-10, PM-2.5 and PM-1.0 stabilized at 12, 8 and 4 μg/m^3^, respectively. After the room ventilation was turned on at 7:00 the concentrations of all aforementioned particle fractions decreased rapidly to about 3 μg/m^3^ at 8:00 h.

**Fig. 6:**
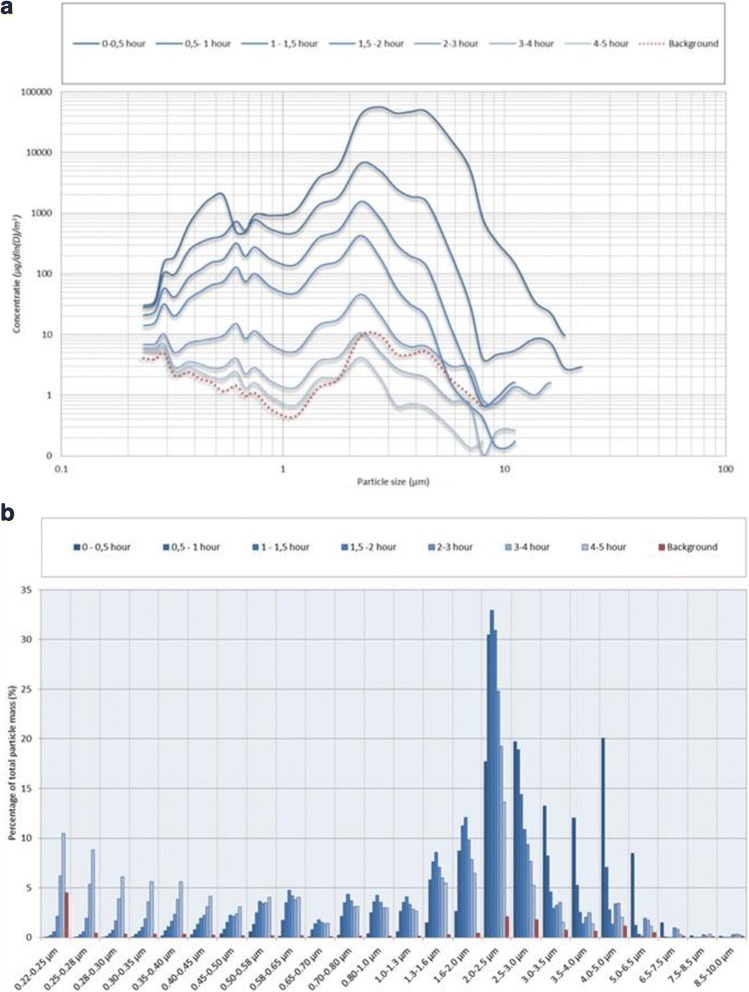
Optical particle size distributions (experiment 2). **a** Particle mass size distribution at time intervals of 30 min. This distribution was recorded in the spray booth during 30 min prior to the start of the spray application (red dotted curve). **b** Relative change of the contribution of each channel to the total measured mass concentration with a time interval of 30 min. The relative contribution of the background to each channel is presented for reference (red bars)

The particle mass size distributions shown in Fig. 6a and b suggest that the particles produced during spraying are of the thoracic size fraction. Due to settling of coarse particles the relative contribution of respirable particles and specifically submicron particles increased over a time frame of 5 h. In the first period after the spray application there is a considerable contribution from particles with a size of about 0.5 μm but they are lost due to evaporation or agglomeration.

The particle concentrations were also measured by filter sampling (results not presented) and show that the time-weighted average concentrations of inhalable, thoracic and respirable dust were high during the first 50 min after the start of the spraying. Over the post-exposure period the concentrations were much lower and approached levels, which corresponded to levels often observed as a normal background for a workplace in a production environment.

### Modelled vapour concentrations

**Fig. 7:**
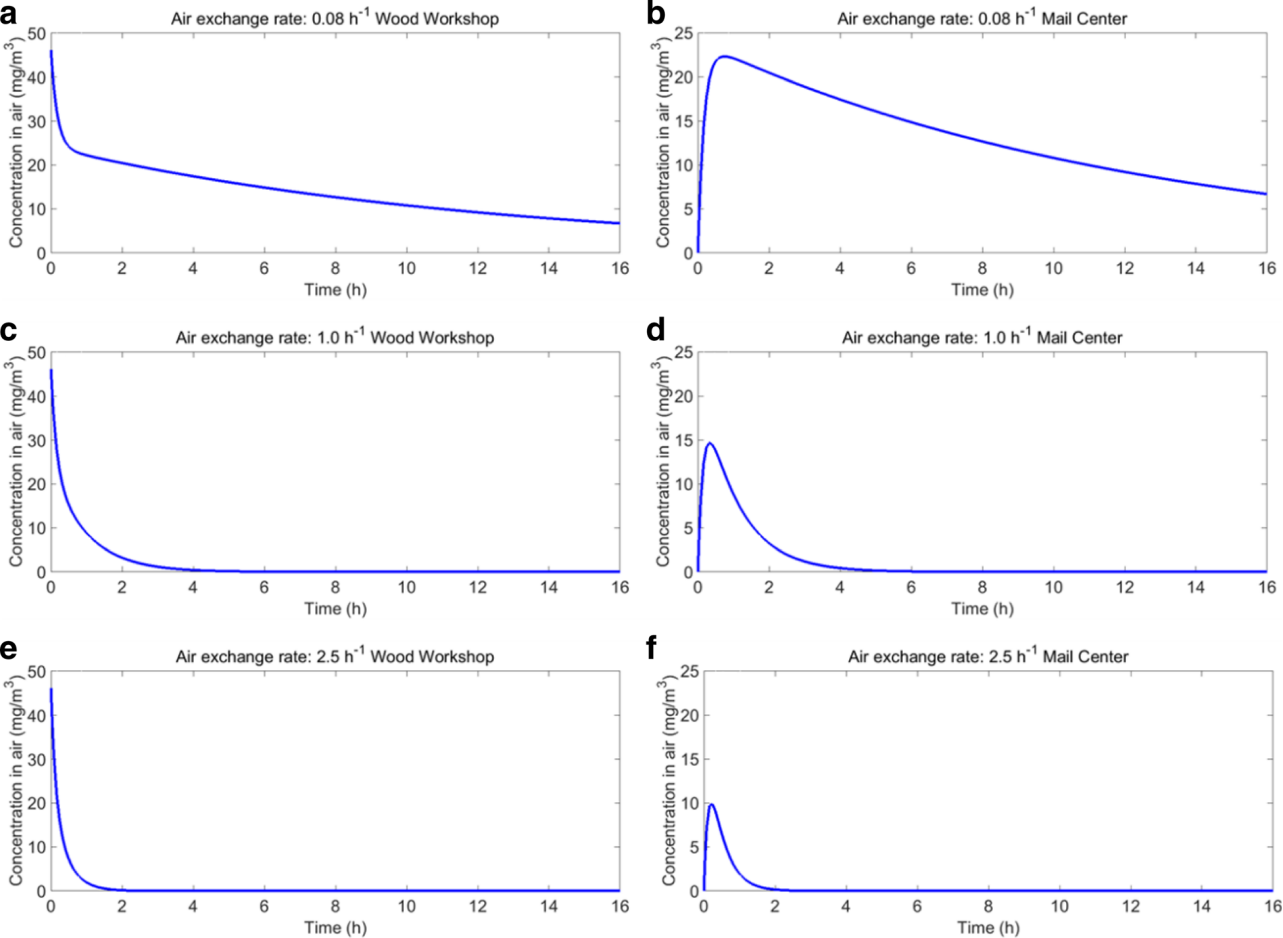
Concentration time pattern for the most abundant VOC (glycol ether) for three scenarios with different air exchange rate for both the wood workshop (**a**, **c** and **e**) and the post sorting room (**b**, **d**, **f**). The assumed air exchange rates were 0.08 h^−1^ (**a**, **b**), 1.0 h^−1^ (**c**, **d**) and 2.5 h^−1^ (**e**, **f**)

In Fig. 7 three scenarios are presented with three different air exchange rates. The concentration time pattern concentration for the most abundant VOC was modelled in the wood workshop and in the mail sorting and distribution centre. In the wood workshop the concentration decreased lognormally. In the mail sorting centre the concentration increased rapidly until a peak is reached within 1-2 h. Then the concentration decreased in a similar way as in the wood workshop. The assumption of the air exchange rate has a profound influence on the concentration in the mail sorting room 15 h following application of the product in the wood workshop. At this time point we estimate a concentration of 7.2, 7.3 × 10^−6^ and 1.2 × 10^−16^ mg/m^3^, corresponding to a respective assumed air exchange rate of 0.08, 1.0 and 2.5 h^−1^.

## Discussion and conclusion

In a recent review of health effects from exposure to waterproofing products (Hays and Spiller, 2014) [31] it is suggested that new formulations of fluroropolymer sprays lead to formation of smaller particles. It is suggested that this is in line with the general understanding that ultrafine particles with a low water solubility have toxic properties [31−33]. Hays and Spiller [31] argued that the increasing particle size of some fluoropolymer fumes was associated with a reduction in toxicity. A smaller particle size will also allow the product to reach deep into the lungs, to the alveoli and respiratory bronchioles that are covered by a thin liquid film of lung surfactant [34, 35]. The product used in the wood workshop has previously been tested in an assay assessing lung surfactant function in vitro and in a mouse bioassay. The breathing pattern of mice exposed to the product was monitored (referred to as “Wood impregnation” in Sørli et al. [17]. The waterproofing product was found to damage the lung surfactant function in vitro. Furthermore, it severely damaged the lungs of mice exposed to “wood impregnation”. This was seen as a rapid and irreversible depression of the tidal volume at an exposure concentration of 39 mg/m^3^ or above.

In our case the product label stated that the product should be rubbed on the surface using a cloth. However, the product label did not contain an explicit warning not to spray the product. The spraying of the product in the wood workshop left the applicant (person A) and his wife (person B) unaffected but caused a serious chemical-induced pneumonia in a mechanic (person C) who entered the workshop ca. 2-3 h later. On the next day (ca. 15 h after spraying) another nine workers in an adjacent room of the building suffered from mild airway symptoms. The prevailing wind direction supported the assumption of transfer of vapours and airborne particles by infiltration from the wood workshop into the mail sorting centre through a 2.0 m^2^ open air connection in the wall. No exposures other than the use of the waterproofing product could be identified to explain the observed health complaints. The labor inspector found no chemicals in the wood workshop and the fire brigade did only detect organic vapours at low levels using a photoionization detector. A psychogenic mechanism in the occurrence of health complaints in the nine mail workers could be ruled out because the employer and the workers of the mail sorting area were unaware of the occupational accident that had occurred in the wood workshop the previous day.

Remarkably, the exposure of persons A-C showed an almost dichotomous response; person C was severely affected by a 30 min exposure with a latency of 2-3 h following the moment of spray application. This is in contrast to the unaffected persons A and B. Aside from the different exposure times, person C was an active smoker, as opposed to persons A and B. The response of the smoker is in accordance with some previously reported cases. For instance, a 25-yrs. old woman developed severe dyspnae, cough and slight fever 5 h after having used a waterproofing spray [8]. Following the spraying, she had smoked a cigarette with spray-contaminated fingers. The thermal degradation products were suggested to cause the severe hypoxemia and leukocytosis that was observed in the clinical examination. CXR and CT showed diffuse infiltration in both lungs and patchy alveolar infiltration. In another case, seven out of 13 workers reported symptoms of cough, fever, chills, aching and weakness after the application of a fluorocarbon polymer. Four of the seven workers reported shortness of breath. Those with symptoms, considered to be polymer fume fever, were all smokers, whereas most of the nonsmokers were without symptoms. The authors considered exposure to pyrolysis products via contaminated cigarettes to cause the symptoms [36]. In a third case, workers developed polymer fume fever after using a mold-release spray containing a polytetrafluoroethylene (PTFE). It was assessed that poor general hygiene and smoking during and after the spraying contributed to the symptoms [37]. A previously healthy 21-year old man who was machining PTFE, presented symptoms that were ascribed to polymer fume fever. The symptoms occurred suddenly while smoking a cigarette 2 h after leaving his workplace. The cigarette was from a pack that had been open and was lying next to his work station [38].

In experiments conducted in 1962 by DuPont, cigarettes were spiked with PTFE in doses ranging from 0.05 to 0.4 mg. Forty volunteers participated in the experiment. It was shown that smoking a cigarette spiked with 0.4 mg of PTFE induced polymer fume fever in 9 out of 10 subjects [39]. In a recent review of the literature, Hays and Spiller [31] conclude that specific precautions such as dermal protection and frequent hand washing is recommended to avoid the formation of pyrolysis products to be formed as a results of poor personal hygiene related to tobacco smoking.

Person C did not wear gloves when performing the repairs in the wood workshop where the waterproofing product had been sprayed. Although person C did not notice any contamination on tools or surfaces and declared not to have been in direct contact with the treated table top, we suggest that it is likely that his hands became contaminated with the waterproofing product due to handling of tools and touching of machinery. We suggest that during the break, person C rolled cigarettes with contaminated fingers. When smoking these cigarettes perfluorinated contamination in the cigarettes became pyrolized, followed by inhalation during smoking. It is a fair assumption that surfaces in the workplace had been covered with the product from a wide deposition in the work environment. This assumption is supported by the rapid decrease of the contribution of particles with a mass median aerodynamic diameter (MMAD) of 2.5 to 6.5 μm in the first half h after the application (Fig. 6b). The soiling of surfaces with the product is likely enhanced by the positive charge of the perfluorinated nanospheres in the product.

A similar case of secondary exposure involved eleven workers with severe dyspnea [17]. These workers had been exposed following an open air discharge of pyrolysis products from fluorocarbon monomers at a distance of 35 m between 20:00 and 0:00. Perfluoroisobutylene, hydrogen fluoride, hexafluoropropylene, tetrafluoroethylene, chlorotrifluoroethylene were detected in the sub mg/m^3^ range, 22 h after the release. Five workers were diagnosed with an acute respiratory distress syndrome (ARDS). One worker died of pneumonia and four surviving workers showed bilateral ground-glass opacities on their initial CT scans and displayed interstitial pneumonia, which gradually improved, similar to our case. No information on exposures to airborne particulate matter was reported. In addition to the direct exposure to polymer fumes, the smoking history and its effects on bronchial or alveolar inflammation or surfactant composition, may also interact with the exposure to the waterproofing product. In similar cases it was seen that exposure to a water repellent product, interacted with underlying idiopathic pulmonary fibrosis, resulting in a fatality [40]. To our knowledge there were no pre-existing health issues that may have contributed to condition of our patient. As he was 40 years old at the time of the event, pre-existing idiopathic pulmonary fibrosis is very unlikely. Furthermore, during his last outpatient visit the chest X-ray demonstrated no remaining abnormalities (a HR-CT scan however was not repeated).

In our case, respiratory symptoms were also reported by the group of postal workers, who were exposed 15 h after application of the product. It is suggested that the draft caused by an outdoor wind speed of 3-4 m/s from the South to South East (see Additional file 1: Table S2 for a full account of the weather report) pushed the overspray cloud of particles from the wood workshop to the mail sorting centre through the 2.0 m^2^ open air connection in the wall separating the two adjacent rooms. Such behavior of waterproofing product aerosols was observed in other studies [23]. Based on our observations in Experiment 2 these particles must have deposited within the hour on tables and floors in the mail sorting centre. On the treated table top the aerosols would merge and polymerize and form a hard top coating. However, in this case the aerosols were dispersed over a wide surface, lost water and remained droplets with polymer and residual fluoro-compounds potentially absorbed on other deposited dust particles. When the workers entered in this work area on the next morning it is likely that when they started working, these deposited particles became airborne through resuspension and were subsequently inhaled. Exposure to coarse dust from hard floors is known to cause particles exposure peaks when they are resuspended from the floor by persons entering an indoor environment [41, 42]. It is suggested that these particles (that were initially derived from the aqueous product) had completely dried out and became water insoluble solid fluoropolymer cores that may have been causative of the effects in the airways as described (see Additional file 1: Figure S7). If the dust particles penetrated deeply into the lungs, they may have inhibited lung surfactant function, causing the workers symptoms. In their case Tan et al. [28] indicated that the pyrolysis products that had been released in open air ‘entered the workshop through ventilation holes on the top of the building’s wall’; thus the workers had been exposed during the night and were then admitted to hospital after their shift (an exact time was not reported) [17]. It was indicated that (similar to our case) the workers were not warned by odor or visual smoke, and they had been exposed for several hours during their shift before symptoms occurred. The first symptoms of dry cough and increasing dyspnea symptoms indicated hospital admittance. There are some similarities with our case, e.g. the setting (smoker entering a work space, no alert, latency in onset of symptoms, type of symptoms); however, an important difference is that Tan et al. reported emissions from pyrolysis products, including hydrogen fluoride and much smaller fluorocarbon monomers as much smaller gas phase components [29], than we identified in our reconstruction [17]. Moreover, both the exposure type and level and the spectrum of health effects, including a fatal outcome, indicate a much more severe incident.

In a recently reported incident a group of 43 persons were exposed to a tile coating product that was applied using a high pressure airless spray gun in a supermarket in Greenland [23]. Of this group 39 were admitted to the hospital in Nuuk for evaluation. The product contained C_9_-C_13_ alkanes and alkylsiloxanes but no fluorinated compounds. Similar to our case most of the victims were exposed in the same building for less than 2.5 h and some far from the primary source of application (on another floor). Some of them entered the building after cessation of the spray event. They were not warned by odor or by irritating properties of the product. In contrast to our case on the day after the use of the product, surfaces in most of the building were visibly contaminated. In this incident half of the victims were workers and most of them smoked. The spray gun operator and two other workers with high exposure had more severe respiratory symptoms with a decreased oxygen saturation and in two bilateral perihilar infiltrates on CXR were observed. The most common health effects were an onset of dry cough (39/39) and shortness of breath (29/39) within 1-12 h after the incident but with normal oxygen saturation.

An interesting observation in our case is the complete lack of health effects in the applicant. The over spray did not cause any complaints. It must be assumed that the freshly sprayed product consisted primarily of coarse aqueous aerosols with 5% of organic substances (consistent with the product formulation, see Additional file 1: Table S1), and, thus, was not causing an airway response.. We suggest that this situation differed from the situation in the mail sorting centre in the sense that in this case the workers inhaled the (presumably much smaller) dried solid water-insoluble fluoro copolymer particles (see Additional file 1: Figure S7).

In conclusion, we have investigated reported health effects occurring ca. 3 and 15 h after use of a wood impregnation product, respectively. We believe that the lung trauma in the by-stander can be explained by inhalation of pyrolysis products from smoking contaminated cigarettes. The secondary exposure was most likely caused by inhalation of fluoropolymers adsorbed on paper dust particles that became airborne after deposition. This product was marketed as a consumer product not intended for spraying. The product label did not contain any safety sentences and did not refer to possible health or environmental risks. Afterwards, the manufacturer changed the label to include a warning not to spray the product. Also the producer informed the authors that the product in its current composition was withdrawn from the market by the end of 2012.

## Additional file

Additional file 1: Supplemental Material. (DOCX 1277 kb)

## Abbreviations

CID: Collision induced dissociation
CRP: C-reactive protein
CT: Computer tomography
CXR: Chest X-ray
FEV1: Forced expiratory volume in one second
FVC: Forced vital capacity
HRCT: High resolution computed tomography
IOM: Institute of Occupational Medicine
NIST: National Institute of Standardization and Testing
SIM: Selected ion monitoring
SOA: Secondary organic aerosol
SVOC: Semivolatile organic compounds
TD-GC-MS: Thermal desorption gas chromatography mass spectrometry
TLCO: Carbon monoxide transfer factor of the lung
VC: Vital capacity
VOC: Volatile organic compounds
