# Supplementary material for: Pulmonary injury associated with spray of a water-based nano-sized waterproofing product: a case study
Source: J Occup Med Toxicol. 2017 Dec 8;12:33. doi: 10.1186/s12995-017-0180-7 (PMC5721695; doi:10.1186/s12995-017-0180-7)
Supplement: Additional file 1: — Supplemental Material. (DOCX 1277 kb) [file 12995_2017_180_MOESM1_ESM.docx]

**Pulmonary injury associated with spray of a water-based nano-sized waterproofing product: a case study**

Paul T.J. Scheepers^a^, Lucie Masen-Poos^b^; Frits G.B.G.J. van Rooy^c^, Arné Oerlemans^a^; Eline van Daalen^a,f^, Robbert Cremers^a,g^, Hera Lichtenbeld^d^, Bonne Biesma^b^, Jorid B. Sørli^e^, Ismo K. Koponen^e^, Søren Thor Larsen^e^, Peder Wolkoff^e^ and Asger W. Nørgaard^e^

^a^ Corresponding author: Research Lab Molecular Epidemiology, Radboud Institute for Health Sciences Radboudumc, PO Box 9101, 6500 HB Nijmegen, The Netherlands; telephone +31 24 3616878; fax +31 24 3613505; e-mail [paul.scheepers@radboudumc.nl](mailto:paul.scheepers@radboudumc.nl); ^b^ Department of Lung Diseases, Jeroen Bosch Hospital, ’s-Hertogenbosch, The Netherlands; ^c^ Arbo Unie Expert Centre for Chemical Risk Management, Utrecht, The Netherlands; ^d^ Nanosafety Consulting, Maastricht, The Netherlands; ^e^ The National Research Centre for the Working Environment, Denmark; ^f^ Present address: Ministry of Social Affairs and Employment, The Hague, The Netherlands’; ^g^ Present address: Witteveen+Bos Consulting, The Hague, The Netherlands; e-mail addresses: hlichtenbeld@gmail.com; f.vanrooy@kpnplanet.nl; l.masen@jbz.nl; b.biesma@jbz.nl; robbert.cremers@gmail.com; eline.vandalen@gmail.com; ikk@nrcwe.dk; soerenthorlasrsen@hotmail.com; pwo@nrcwe.dk; asgerngaard@gmail.com; arne.oerlemans@radboudumc.nl

Abbreviations: AFM, atomic force microscopy; ATR, attenuated total reflectance; DLS, dynamic light scattering; EDX, energy dispersive X-ray; ED-XRF, energy dispersive X-ray florescence; EO, ethylene oxide; FTIR, Fourier transform infrared spectroscopy; NMR, nuclear magnetic resonance; PMDS, polydimethylsiloxane; PO, polyethylene oxide; TEM, transmission electron microscopy (TEM) with, WD-XRF, wavelength dispersive X-ray fluorescence

Introduction

Waterproofing products are normally used to make textile fabric (clothes, tents) or leather (shoes) water resistant. There are also applications of similar products for solid surfaces such as e.g. floor tiles and wood. In the literature such products are described by their field of application (e.g. ‘antirain spray’) or by their chemical composition (‘fluor carbon products’). The performance characteristics of such products are described by the ‘lotus effect’ mimicking the water-repellent nature of the leaves of the lotus plant (Neinhuis and Barthlott, 1997) which is described as ‘superhydrophobic’ and attributed to characteristics of natural nano-materials (Koch et al., 2010).

The conventional organic solvent-based waterproofing products and the newly developed water-based products can be applied on surfaces by rubbing, using a cloth or by spraying. Here we restricted ourselves to spray applications and health consequences of inhalation of the generated aerosols. In consumer applications such aerosols are usually generated at atmospheric pressure by a spray pump or from a canned product using a propellant, whereas professional users apply such products often using air pressure paint spray equipment.

The traditional products used for waterproofing purposes contain three types of components: a water proofing agent or repellant, a solvent and a propellant. In products used for the American market before 1990, the active ingredient was most often an aliphatic fluororesin or silicon resin, phthalate or wax (Figure 1). The solvent component was most often an aliphatic hydrocarbon (heptane), cyclic hydrocarbon (cyclohexane) or chlorinated hydrocarbon (1,1,1-trichlorothane) but was not critical in the final result because the solvent evaporates during and after the spray process. In a canned product, *n*-propane, isopropane and/or *n*-butane are often used as propellant. If the product is available with a spray pump, no propellant is needed. With the Clean Air Amendment of 1990, in the US, the use of solvents like chlorinated hydrocarbons was prohibited (Testud et al., 1998, Burkart et al., 1996; Wallace et al., 2005). This caused the replacement of fluoroalkane resins by fluoroalkenes in second generation products that were better soluble in aliphatic hydrocarbon solvents (Hubbs et al., 1997; Testud et al., 1998; Daubert et al., 2009). A more recent development is the change from an organic solution to a water-based suspension. In this recent change, fluoridated block copolymers were introduced as active ingredients. The properties of these products were optimized using different types of detergents and surfactants to stabilize the suspended polymers in water and improve surface properties as a water-repellent.

Over the past 18 years, different health effects of the use of waterproofing agents have been described in different reports involving over 200 individuals (Woo et al., 1983; Laliberté et al., 1995; Burkhart et al., 1996; Testud et al., 1998; Tatino et al., 1999; Tagawa et al., 2003; Bonte et al., 2003; Lazor-Blanchet et al., 2004; Heinzer et al., 2004; Wallace et al., 2005; Vernez et al. 2006; Kobayashi et al., 2006; Hasimoto et al., 2006; Daubert et al., 2009). These cases describe mostly local effects on the airways and dyspnea with (dry cough). Overall it can be concluded that a majority of thecomplaints resulted from a response in the upper airways, leading to dyspnea and in some cases subsequent tachypnea, tachycardia and mild cyanosis.

Nanoparticles may enhance the technical performance of these products but might also enhance the physiological response if finely dispersed particles become and remain airborne during application and are subsequently inhaled. It has been shown that nano particles can penetrate deep into the non-ciliated alveolar part of the lungs (Schmidt et al., 2009; Oberdörster 2001), reaching alveoli where they can interfere with lung surfactant or induce inflammation.

Methods

One liter of the product was acquired directly from the manufacturer in the packaging that was also used for delivery to retailers and end-users. The packaging consisted of a white polyethylene bottle with screw cap. The label indicated the name of the product, instructions for use and a statement that it was water-based. Concerning the purpose and result of its use, it was stated that it would make wooden surfaces water and dirt repellent and provide almost invisible protection that would prevent stains from food or beverage. The product was advertised for surface treatment of hardwood furniture (for both indoor and outdoor use). The label did not contain any symbols referring to possible hazards to health or to the environment but the product contained the following phrases (translated from Dutch/French): ’Keep out of range of children; prevent direct contact with skin and eyes; use only in a well-ventilated area; store in closed bottle; do not induce vomiting if swallowed and consult a physician and show the package and label; we do not accept liability in the case of wrong use; attention: store frost-free.’ The product was advertised as a water-based and solvent free product. The label did not contain any further information on the composition. No reference was made to the product containing nanoparticles or nanotechnology. The producer provided information about the formulation of the product (Table 1).

*Physico-chemical characterization*

All analytical characterization was performed at the Nano Characterization Lab of DSM R&D Solutions, Royal DSM N.V. (Geleen, Netherlands). The product was characterized by dynamic light scattering (DLS) transmission electron microscopy (TEM) with energy dispersive X-ray (EDX) analysis, atomic force microscopy (AFM), Fourier Transform Infrared Spectroscopy (FTIR) and attenuated total reflectance (ATR) FTIR spectroscopy, Raman Spectroscopy, Wavelength Dispersive X-Ray Fluorescence (WD-XRF), Energy Dispersive X-Ray Fluorescence (ED-XRF), ^1^H-nuclear magnetic resonance (NMR) spectroscopy and ^13^C-NMR spectroscopy.

Results

*Chemical characterization of the product*

The pH of the original sample was 5.9 and 6.0 after 50-fold dilution in 1 mM KCl solution. A surface charge of +31.3 mV was determined by Zeta-potential.

The median hydrodynamic radius or Z-average determined by DLS was 71 nanometer (Figure S2). AFM images suggest a slightly larger median diameter of 73 nm and showed that the particles have a spherical aspect (see Figures S1-S2). The particles appeared to have a solid core of approximately 60 nm, consisting mostly of organic silica (no water observed). From the core to most outer shell the structure density of the particle decreased gradually (as observed by diminishing energy dissipation). The shell consisted of fluor-containing polymers with traces of sulfur and possibly some calcium as determined by ED-XRF and WD-XRF.

EDX-TEM analysis confirmed the particle size as determined by AFM and DLS. TEM images of undiluted product showed how the sample was dispersed on the grid (Figure S2). The sample ingredients formed a thin coating with slightly thicker and thinner blobs. Most particles appeared to be composed of organic silicates. EDX measurements of these particles showed no inorganic elements (Figure S6). By AFM, beside the silicone particles, also darker particles of probably inorganic nature were detected (cf. Figure S3). EDX of these particles revealed the presence of sulfur, aluminum and iron as traces.

FT-IR and RAMAN spectra of the coating suggested presence of ethylene oxide (EO), fluorinated aliphatic compounds and organic silicones, presumably polydimethylsiloxanes (PDMS). Also an ester functionality was observed in the residual ATR-FTIR spectrum (peak at 1 736 cm^-1^).

Further analysis of the samples was performed by ^1^H NMR and surface analysis. This showed that the organic components were a mixture of surfactants, hydrophilic and hydrophobic (block co)polymers containing EO and propylene oxide (PO, Figure S4). Si(CH_3_) groups were observed as relative broad signals which supports the earlier finding of PDMS. The amount is roughly estimated to be less than 10 % (excluding water). A series of characteristic peaks were observed, most of the sharp resonances belonging to a polyether structure consisting of possibly (partially) methylated polyethylene oxide (pEO) and polypropylene oxide (pPO). The EO/PO ratio was calculated to be approximately 1:3. This may indicate a triblock pPO polymer, end-capped with (a few) EO groups.

A more detailed study was performed to establish the ^13^C chemical shifts by two dimensional nuclear magnetic resonance spectroscopy (2D NMR). This analysis provided more detailed information about the EO/PO sequences. In the 2D gHSQC spectrum, the spectral region between 50 and 80 ppm chemical shift, represents all characteristic signals corresponding to O-CH, OCH_2_ and OCH_3_ moieties (Figure S5). Block co-polymer tails were estimated to have molecular weights below 1 000 Da. From this spectrum we also deduced that EO and PO were present within the same molecules and consist of various sequences ending at CH(CH_3_)-OH, CH_2_-OH and CH_2_-O-CH_3_. The average molecular weight appears very low as many individual signals could be detected.

Discussion

*Physico-chemical characterization*

The particles in the product were analyzed using different spectroscopy and microscopy techniques. Combining this information, we can conclude that the product contains solid spheres consisting of water-free solid organic silica cores, with a soft shell consisting of aliphatic hydrocarbons characterized as tri-block-copolymers pEO and pPO, end-capped with EO. Apart from carbon, hydrogen and fluor the particles appeared to contain sulfur, aluminum and iron contaminants in the product. The particles appeared to have a spherical aspect and form a stable suspension in water showing a slight positive charge, strong hydrophobic properties and a negligible tendency to form clusters in the undiluted product as well as in the fifty fold diluted KCl solution. The mass median diameter of the spheres was observed to be 71 to 73 nm as determined using different measuring principles.

*Explaining health effects by chemical composition*

The by-standers in the primary exposure and also the persons involved in the secondary exposure on the next day, did not observe any spray mist or smell. Furthermore, a role of organic solvents is not likely because of the composition of the product and lack of finding of enhanced levels of organic vapors on the incident location, based on measurement by use of a photo ionization detector with a detection limit in the low ppm range for hydrocarbon vapors.

In most previous reports an active fluororesin ingredient was implicated as the agent responsible for an acute pulmonary response (Burkhard et al., 1996; Testud et al., 1998; Hubbs et al., 1997; Lazor-Blanchet, et al., 2004; Daubert et all, 2009). Cleaning and sealing sprays such as “Magic Nano” and “Finy” were the first products suspected of health effects in humans, referring to the use of nanotechnology as a possible contributing factor by its toxicity. However, data supplied by the producer showed that these products in fact did not contain nanoparticles and the health effects were presumably caused by other properties, in particular the mist delivered by the spray mechanism (DG Health and Consumer Protection, 2004). It was suggested that after evaporation of the solvent, semivolatile fluorosilanes settled on the surface of particles that were presumably formed by an (unspecified) corrosion inhibitor. These particles could penetrate into the alveolar region of the lung due to their size distribution characteristics. It was also suggested that changes in the surfactant function of the lung lining fluid subsequently gave rise to the observed pulmonary edema (Pauluhn et al., 2008). It is important to note that the product did apparently not contain nanoparticles by itself and it was suggested that respirable (but not necessarily nano-sized) particles were formed during spraying of the product. This case has been recognized as a classical example where the authorities, scientific community and the public was misled by suggestions in the media that the health effects were somehow related to the application of nanotechnology in this product (Zimmer et al., 2010).

Song and co-workers published a clinical report, suggesting the involvement of nano-siezed polyacrylate particles in an occupational exposure of 5-13 months in seven female workers of a print production facility in China (Song et al., XXXX). A product that contained a paste material polyacrylic ester and a range of organic constituents were automatically air sprayed on a polystyrene board by three atomizing air nozzles at a pressure of 100-120 kPa. The coating was heat-cured at 75-100 °C, which produced smoke. Exposures were not measured but suggested to be high because the spray operation and heating occurred in a small confined space and exhaust ventilation was reported to be dysfunctional for 5 months. Workers were exposed during loading of the paste material, spraying and drying during 8-12 h/day and did not use personal protective equipment except for a cotton gauze mask. The seven workers were hospitalized and reported no history of disease and reported no history of smoking. Two of the workers died in the hospital. Lung pathology indicated non-specific inflammation, fibrosis and foreign body granulomas in pulmonary pleura. Nano-sized particles were observed by TEM intracellular in lung epithelial and mesothelial cells and extracellular in chest fluid. A range of organic solvents (butanoic acid, butyl ester, *n-*butyl ether, acetic acid, toluene, di-*t*-butyl peroxide, 1-butanol, acetic acid ethenyl ester, isopropyl alcohol and ethylene dioxide that were characterized in the paste material by GC-MS, but the polyacrylic ester precursor material was not analyzed. This study suffered from a number of limitations, e.g. no electroscope images of the ~30 nm nanoparticles were shown, no evidence of nanoparticles in the workplace dust and no chemical analysis was carried out to confirm that the particles that were found in the lungs or on the workplace were indeed polyacrylic particles (Maynard, 2009; Ross 2009). In comments that were published on the internet experts in the field suggest that it is difficult to rule out the possibility that other work-related exposures may have caused the inhalation trauma. Some of the commentaries indicate that a causal relationship between the exposure and the observed lung pathology cannot by scientifically justified (Maynard, 2009). The exposure reported by Song and co-workers clearly involved polymer fume particles and a range of (toxic) organic fumes. Also the exposure was to freshly-generated process emissions which include new compounds from thermodegradation. This is in contrast to the exposure that is described in the present study, which describes an exposure lacking a heat source, involves solid particles of different physicochemical nature and also involves much lower exposure to both particles and organic vapors.

*Explaining health effects from physical properties*

Upon formation of aerosols, the total number of particles that can be inhaled is potentially very large. Nanoparticles contribute very little to overall mass but may reach very high concentrations if expressed in number or surface units, instead of mass units (Oberdörster, 2001). In the product we studied, fluoroalkylethylacrylate co-polymers were used to modify surface properties and may have increased or activated the surface of the silicon spheres. An important property of the fluoroalkylethylacrylate co-polymer active ingredient (either fluoroalkane, fluoroalkene or fluor acrylate co-polymer) is its poor water solubility which may cause prolonged interaction with lung tissue if spray particles are not cleared from the lungs (e.g. due to high deposition efficiency of such insoluble particles in periphery of the airways where clearance is low due to lack of cilia).

We suppose that when air spraying the product most of the course liquid aerosols will readily settle on the surface which is treated due to gravity. However, finer liquid aerosol from the side stream of the jet spray nebula may remain airborne for a much longer time and it is expected that the lipophilic fluoroalkylethylacrylate nanoparticles may show anisotropy at the water-air interface (Figure 6). As long as the particles remain airborne, water may be lost due to evaporation, decreasing the sphere volume until an equilibrium is reached leaving solid particles in the breathing zone of a bystander appearing on the work floor hours later. It is suggested that the particles form solid polymer clusters in the same way as the fluoroalkylethylacrylate active ingredient forms a hard-solid water-repellent polymer network on the treated surface. It is supposed that these networks of co-polymers may also be formed while the fine spray aerosol is airborne due to the loss of water and that the side spray aerosol reaches a stable particles size which may be respirable, possibly submicron and remain airborne for a long time. This would explain that workers could be exposed in an adjacent room on the following day. Similar behavior of 75 nm size TiO_2_ particles was described by Chen and co-workers (2010). Specifically particles of 0.1-0.5 µm tend to have the lowest deposition rates in a low-ventilation environment (Lia and Ahmadia, 1992; Xu et al., 1994), increasing the probability of movement with air from one room in a building to another (Liu et al., 2001; Miller and Nazaroff, 2001). Once in the breathing zone such particles will readily penetrate into the airways and reach the alveoli (Schmidt et al. 2009).

Most of the previously published reports of health effects of waterproofing products suggest that health complaints arise from inhalation of spray particles. The complaints suggest spray particles have an irritating effect on the upper airways but also indicate a possible effects in the proximal airways. Some studies suggest that the particle size and number characteristics of inhaled sprays could have a role in explaining acute pulmonary toxicity (Testud, 1995; Yamashita et al. 1997a; 1997b; Vernez et al., 2006).

In attempts to characterize the physical properties of the product we did not find any indication of the particles becoming coagulated or aggregated. On the contrary, we observed that the majority of particles remained singularly and finely dispersed in the aqueous environment. Inhaled water-based spray particles are, once deposited, anticipated to merge with lung lining fluid, releasing numerous finely dispersed nano-size spheres particles that could interact with lung tissue. This could potentially lead to a very high dose in terms of particle numbers and surface (Oberdörster, 2001). This may explain why in this case particles still caused an adverse reaction, even though the concentrations were presumably extremely low by dilution in a large indoor volume of air over a relative long period of time (approximately 16 hours).

*Policy implications*

The present case describes a situation which could be categorized as ‘unintended use’ of this consumer product since the product label gave instructions to use a cloth to rub the product on the surface. However, the case as it is described could also be interpreted as ‘reasonably foreseeable misuse’ of the product. The label did not contain a warning not to spray the product or use it exclusively outdoor. If the involvement of the silicone particles with fluororesin coating could be confirmed in toxicity studies, the product would have to be labeled as ‘toxic’.

In April 2011 one of the authors (PS) was informed that the product label was changed to include an explicit warning ‘Do not spray this product’. Also the producer sent out an alert to professional users to inform them about the possible health risk of inhalation of spray mist based on the particular incident described in this paper. The producer also announced the phasing out of active ingredient which was not notified for use in the European Union as part of REACH requirements. The producer plans to find a substitute for the active ingredient.

Acknowledgements

The authors would like to thank the producer for sharing technical information on the waterproofing product.

References

D.S. Alberts, D.J. Garcia. Drugs, 54 (1997) 30-35.

K.K. Burkhart, A. Britt, G. Petrini, S. O'Donnell, and J.W. Donovan Toxicol. Clin. Toxicol., 34 (1996) 21-24.

Bundesinstitut für Risikobewertung (BfR) (2009), Protokoll vom 27./28. April 2009 available on <http://www.bfr.bund.de/cm/207/2_sitzung_der_bfr_kommission_bewertung_von_vergiftungen.pdf>

Chanan-Khan A, Szebeni J, Savay S, Liebes L, Rafique NM, Alving CR, Muggia FM. (2003) Complement activation following first exposure to pegylated liposomal doxorubicin (Doxil): possible role in hypersensitivity reactions. Ann Oncol. 14:1430-1437.

B.T. Chen, A. Afshari, S. Stone, M. Jackson, D. Schwegler-Berry, D.G. Frazer, V. Castranova, and T. A. Thomas. Inhal Toxicol. 22 (2010) 1072-1082.

D. Christmas. Anaesthesia, 39 (1984) 470-473.

G.P., Daubert, H., Spiller, B.I. Crouch, S. Seifert, K. Simone, S. Smolinske. Med. Toxicol., 5 (2009) 125-129.

Directorate General Health and Consumer Protection (2006) Latest safety news - Cleaning and sealing sprays. <http://ec.europa.eu/consumers/safety/news/nanosprays_en.print.htm>

D. Dye, and J. Watkins Br Med J., 280 (1980) 1353.

D.G. Ebo, G.C. Piel, V. Conraads, W.J. Stevens. Ann Allergy Asthma Immunol. 87 (2001) 243-245.

R. Heinzer, V. Ribordy, B. Kuzoe, R. Lazor, and J.W. Fitting J.W. Thorax 59 (2004) 541-542.

A.F. Hubbs, V. Castranova, J.Y. Ma, D.G. Frazer, P.D. Siegel, B.S. Ducatman, A. Grote, D. Schwegler-Berry, V.A. Robinson, C. Van Dyke, M. Barger, J. Xiang, J. Parker, Toxicol. Appl. Pharmacol., 143 (1997) 37-46.

ISO (2008) Nanotechnologies ― Terminology and definitions for nano-objects ― Nanoparticle, nanofibre and nanoplate (ISO/TS 27687:2008). <https://www.astandis.at/shopV5/Preview.action;jsessionid=064DF00C45FFD67CA46BC12A3F61862F?preview=&dokkey=352631&selectedLocale=en>

K. Kobayashi, S. Tachikawa, T. Horiguchi, R. Kondo, M. Shiga, M. Hirose, Y. Sasaki, H. Torigoe. Nihon Kokyuki Gakkai Zasshi 44 (2006) 647-652.

K. Koch, B. Bhushan, W. Barthlott, Functional plant surfaces, smart materials. Handbook of Nanotechnology, third ed., Springer, Heidelberg, Germany, 2010.

Maynard A. (2009) Nanoparticle exposure and occupational lung disease – six expert perspectives on a new clinical study. 2020 Science. A clear perspective on emerging science and technology. <http://2020science.org/2009/08/18/nanoparticle-exposure-and-occupational-lung-disease-six-expert-perspectives-on-a-new-clinical-study/>

C. Moon, C.F. Verschraegen, M. Bevers, R. Freedman, A.P. Kudelka, J.J. Kavanagh. Anticancer Drugs., 11 (2000) 565-568.

C. Lazor-Blanchet, S. Rusca, D. Vernez, R. Berry, E Albrecht, P.O. Droz, M. A. Boillat. Int. Arch. Occup. Environ. Health, 77 (2004) 244-248.

Laliberté, M., Sanfaçon, G. & Blais, R. Acute pulmonary toxicity linked to use of a leather protector. Ann Emerg Med 25, 841-844 (1995).

Lia, A. and Ahmadia, G. (1992) Dispersion and deposition of spherical particles from point sources in a turbulent channel flow. Aerosol Science and Technology. 16:209 - 226

Liu K.S., Alevantis L.E. and Offermann F.J. (2001) A survey of environmental tobacco smoke controls in California ofce buildings. Indoor Air, 11, 26-34.

Losert S, von Goetz N, Bekker C, Fransman W, Wijnhoven SW, Delmaar C, Hungerbuhler K, Ulrich A. Human exposure to conventional and nanoparticle--containing sprays-a critical review. Environ Sci Technol. 2014 May 20;48(10):5366-78. doi: 10.1021/es5001819. Epub 2014 May 12. Review.

Miller S.L. and Nazaroff W.W. (2001) Environmental tobacco smoke particles in multizone indoor environments. Atmospheric Environment, 35, 2053-2067

Neinhuis C., Barthlott W. (1997): Characterization and distribution of water-repellent self-cleaning plant surfaces. Annals of Botany 79 (6), 667-677

Oberdörster, G. Pulmonary effects of inhaled ultrafine particles. Int. Arch. Occup. Environ. Health (2001), 74: 1-8)

Pauluhn J, Hahn A, Spielmann H. (2008) Assessment of early acute lung injury in rats exposed to aerosols of consumer products: attempt to disentangle the "Magic Nano" conundrum. Inhal Toxicol. 2008 Nov;20(14):1245-62.

Ross BL (2009) Linking Nanoparticle Exposure to Pulmonary Fibrosis and Mortality, Evaluating the Key Messages of Song et al. SAFENANO Europe’s centre of excellence on nanotechnology hazard and risk. <http://www.safenano.org/KnowledgeBase/CurrentAwareness/FeatureArticles.aspx>

Scheepers PTJ, Masen-Poos L, Van Rooy FGBGJ, Van Daalen E, Cremers R, Lichtenbeld H, Biesma B Spray application of a water-based nano-sized waterproofing product involved in pulmonary injury. Submitted.

Schmid O, Möller W, Semmler-Behnke M, Ferron GA, Karg E, Lipka J, Schulz H, Kreyling WG, Stoeger T. Dosimetry and toxicology of inhaled ultrafine particles. Biomarkers. 2009 Jul;14 Suppl 1:67-73. Review.

Song Y, Li X, Du X. Exposure to nanoparticles is related to pleural effusion, pulmonary fibrosis and granuloma. Eur Respir J. 2009 Sep;34(3):559-67. Epub 2009 Aug 20.

Testud, F., Gabrielle, L., Paquin, M.L. & Descotes, J. [Acute alveolitis after using a waterproofing aerosol: apropos of 2 cases]. Rev Med Interne 19, 262-264 (1998).

Uziely B, Jeffers S, Isacson R, Kutsch K, Wei-Tsao D, Yehoshua Z, Libson E, Muggia FM, Gabizon A. Liposomal doxorubicin: antitumor activity and unique toxicities during two complementary phase I studies. J Clin Oncol. 1995 Jul;13(7):1777-85.

Vernez, D.S., Droz, P., Lazor-Blanchet, C. & Jaques, S. Characterizing emission and breathing-zone concentrations following exposure cases to fluororesin-based waterproofing spray mists. J Occup Environ Hyg 1, 582-592 (2004).

D. Vernez, R. Bruzzi, H. Kupferschmidt, A. De-Batz, P. Droz, R. Lazor. J. Occup. Environ. Hyg. 3 (2006) 250-261.

Volcheck GW, Van Dellen RG.Anaphylaxis to intravenous cyclosporine and tolerance to oral cyclosporine: case report and review. Ann Allergy Asthma Immunol. 1998 Feb;80(2):159-63.

Wallace, G.M.F. & Brown, P.H. Horse rug lung: toxic pneumonitis due to fluorocarbon inhalation. Occup Environ Med 62, 414-416 (2005).

Woo, O.F., Healey, K.M., Sheppard, D. & Tong, T.G. Chest pain and hypoxemia from inhalation of a trichloroethane aerosol product. J. Toxicol. Clin. Toxicol 20, 333-341 (1983).

Xu M.D., Nematollahi M., Sextro R.G., Gadgil A.J. and Nazaroff W.W. (1994) Deposition of tobacco smoke particles in a low ventilation room. Aerosol Science and Technology, 20, 194-206.

M. Yamashita, J. Tanaka, M. Yamashita, H. Hirai, M. Suzuki, H. Kajigaya, Vet Hum Toxicol 39 (1997) 71-74.

M. Yamashita, M. Yamashita, J. Tanaka, H. Hirai, M. Suzuki, H. Kajigaya, Vet. Hum. Toxicol. 39 (1997) 332-334.

Zimmer, R., Hertel, R., Göl, G.-F. (eds) Risk Perception of Nanotechnology – Analysis of Media Coverage. Federal Institute for Risk Assessment BfR, Berlin, 183 pp. <http://www.bfr.bund.de/cm/350/risk_perception_of_nanotechnology_analysis_of_media_coverage.pdf>.


Table S1: Timeline with relevant dates and times for workers of primary exposure (A-C) and secondary exposure (D-L).

| Date | Activity | Person | Primary – wood workshop | Secondary – post sorting center |
| --- | --- | --- | --- | --- |
| Wed-Mar-24^th^ | Spray application | A | 17:00 – 17:10 | - |
|  | Post-spray exposure | B | 18:15 – 18:30 | - |
|  | Post-spray exposure | C | 19:45 – 19:55 and 20:30-20:45 | - |
|  | Smoking | C | 22:00 | - |
|  | Admittance to hospital | C | 23:30 | - |
| Thu-Mar-25^th^ | First CXR | C | 02:00 | - |
|  | Second CXR and HRCT | C | 09:30 | - |
|  | First postmen enter | D-L | - | 07:45 |
|  | All workers have symptoms | D-L | - | 10:30 |
| Mar-26-28^th^ | Fatigue and weakness | D-L | - | 2 d |
| Mar-31^st^ | Full recovery | D-L | - | 7 d |
| Mar-29^th^ | Dismissed from hospital care | C | 5 d | - |
| Apr-7^th^ | Full recovery | C | 14 d | - |

HRCT = high resolution computer tomography; CXR = chest X-ray radiography

Table S2: Outdoor weather conditions at the time of the incident at a military airbase Volkel 80 km south of the incident location (source: <http://www.knmi.nl/klimatologie/daggegevens/index.cgi> )

| Date | T (min-max)  (°C) | RH  (%) | Precipitation (mm) | Wind direction  (°) | Wind speed (h max)  (m/s) |
| --- | --- | --- | --- | --- | --- |
| 24-03-2010 | 13.0 (6.1 - 19.7) | 65 | 0 | 130 (SE) | 3.0 (5.0) |
| 25-03-2010 | 14.8 (8.6 - 20.7) | 63 | 2.5 | 170 (S) | 4.1 (7.0) |

Figure 1: Schematic of the setting showing the wood workshop were the initial exposure was caused by the spray application and the adjacent mail sorting/distribution center where health complaints occurred on the next day. There was an open air connection of 0.010 x 20 m between the two rooms.


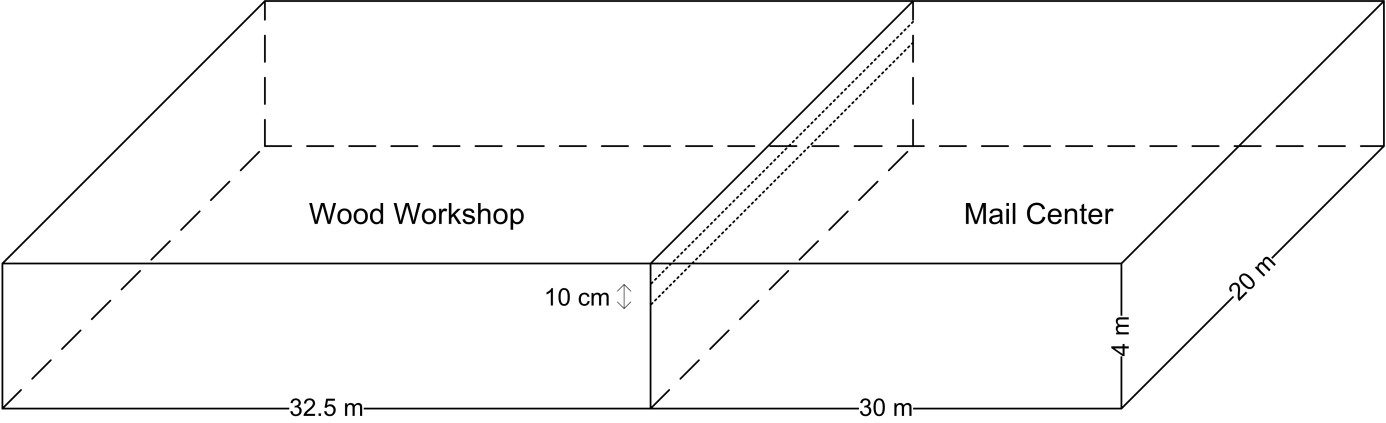


Figure S2: Timeline of changes in solvent of waterproofing products.

| *1st generation* |  | *2nd generation* |  | *3rd generation* |
| --- | --- | --- | --- | --- |
|  |  |  |  |  |
| Aliphatic  Aromatic  Hagolenated Carbons |  | Non-halogenated aliphatic hydrocarbons |  | Water with organic an inorganic additives |
|  | 1990 |  | 2000 |  |

Figure S3: Transmission Electron Microscopty (TEM) image of the dried product**.**

*
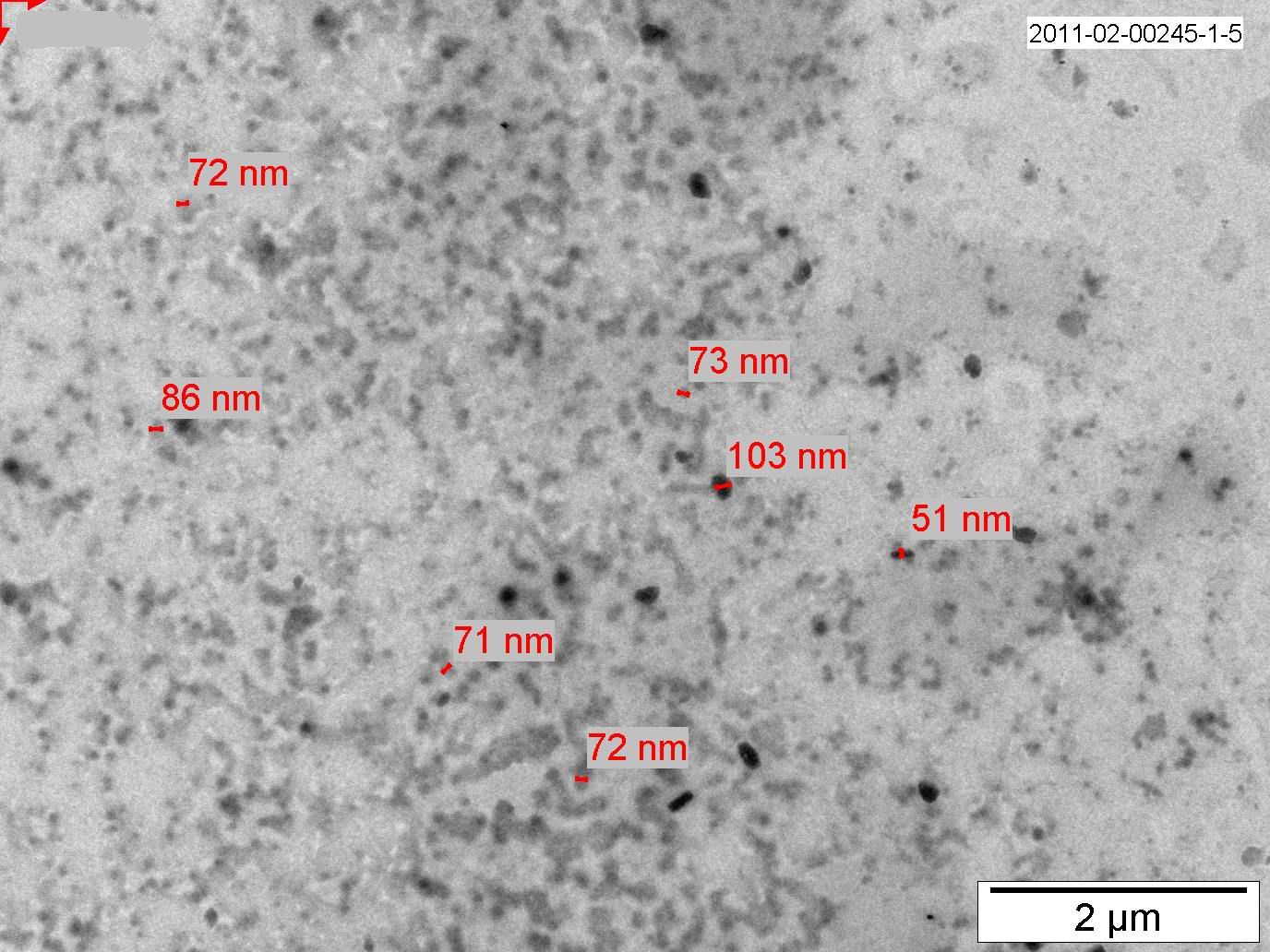
*

Figure S4: Topology (left) and amplitude (right) of particles as measured by AFM (Scale bar 0 -2 μm).


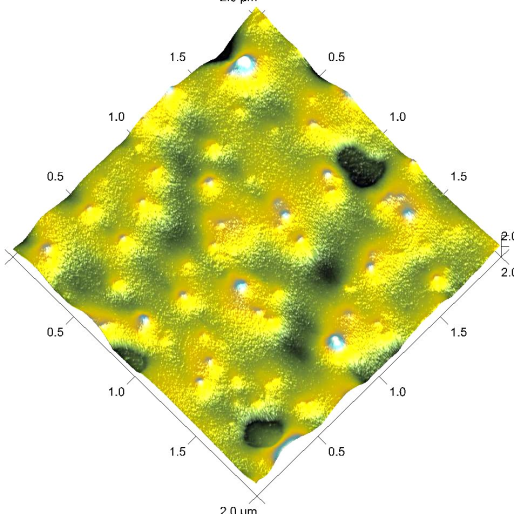

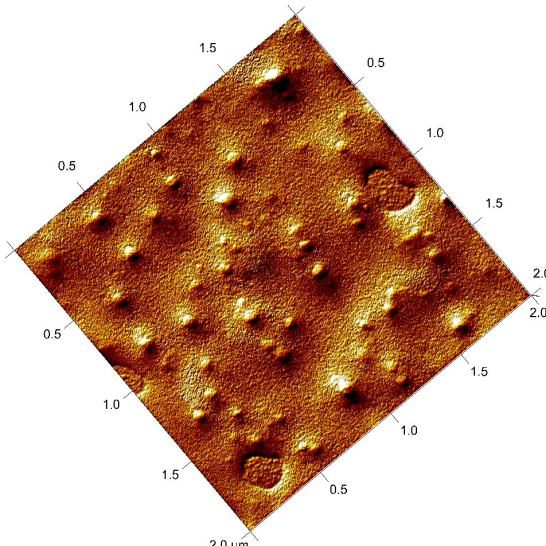


Figure S5: ^1^H NMR spectrum of which the water signal is removed by selective irradiation.

FIgure S6: 2D NMR CH correlation spectrum showing chemical shifts in ppm with blue-purple color corresponding to CH2 moieties and orange-yellow color representing CH3 and CH moieties.

Figure S7: Suggested aging pattern to explain formation of submicron solid clusters of fluoro alkylethyl acrylate co-polymers from side-spray aerosols consisting of aqueous liquid suspensions of the product. Applicant is suggested to exposed to non-respirable spray aerosol of 5-10 µm (a), bystanders were presumably exposed to aged respirable spay particles (1-5 µm) and on the next day workers in an adjacent workplace are assumed to be exposed to submicron solid particles of < 1 µm (Losert et al., 2014).

1. (b) (c)
